# Qualitative Analysis of the Helical Electronic Energy of Inherently Chiral Calix[4]arenes: An Approach to Effectively Assign Their Absolute Configuration

**DOI:** 10.3390/ijms15069844

**Published:** 2014-06-03

**Authors:** Shuang Zheng, Ming-Liang Chang, Jing Zhou, Jing-Wei Fu, Qing-Wei Zhang, Shao-Yong Li, Wei Qiao, Jun-Min Liu

**Affiliations:** 1 Tianjin Key Laboratory on Technologies Enabling Development of Clinical Therapeutics and Diagnostics (Theranostics), School of Pharmacy, Basic Medical Research Center, Tianjin Medical University, Tianjin 300070, China; E-Mails: zhengs0929@gmail.com (S.Z.); changmltmu@gmail.com (M.-L.C.); zhoujing@tmu.edu.cn (J.Z.); fujw@tmu.edu.cn (J.-W.F.); zhangqingwei@tmu.edu.cn (Q.-W.Z.); 2 MOE Laboratory of Bioinorganic and Synthetic Chemistry/KLGHEI of Environment and Energy Chemistry, State Key Laboratory of Optoelectronic Materials and Technologies, School of Chemistry and Chemical Engineering, Sun Yat-Sen University, Guangzhou 510275, China

**Keywords:** inherently chiral, Calix[4]arene, helix theory, absolute configuration

## Abstract

For all microhelices on aromatic rings of inherently chiral calix[4]arene, an expression was derived from one approximation and one hypothesis on the basis of the electron-on-a-helix model of Tinoco and Woody as follows: 
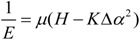
, where *µ* = 1 for the right-handed microhelix and *µ* = −1 for the left-handed microhelix; and *H* and *K* are constant and greater than zero. The expression correlates microhelical electronic energy (*E*) with the atom polarizability difference (Δ*α*) on both microhelix ends, which intuitively and clearly shows the impact of helical substituent polarizability on helical electronic energy. The case analysis almost entirely proves that the qualitative analysis of the helical electronic energy of inherently chiral Calix[4]arenes with the expression is scientific and can be used to effectively assign their absolute configuration.

## 1. Introduction

Inherently chiral calixarenes, whose chiralities result from the dissymmetric substitution of achiral residues on calixarene skeletons, are a type of attractive chiral molecule, because of their potential applications in chiral recognition and asymmetrical catalysis [1,2,3,4,5,6]. Although the first example of inherently chiral calixarene [7] appeared in 1982 and all varieties of them have been reported by now [1,2,3,6], only a few of them have been characterized by absolute configuration assignment, which seriously impedes their application in chiral recognition [8,9,10,11,12,13,14] and asymmetrical catalysis [10,11,15,16,17,18,19,20]. Therefore, the development of effective approaches for the absolute configuration assignment of inherently chiral calixarenes is of great importance.

A few approaches have been used to assign the absolute configuration of inherently chiral calixarenes, including chemical interconversions [19,21,22,23,24,25], X-ray crystallography [23], circular dichroism (CD) analysis [25] and density functional theory (DFT) calculation [26]. However, chemical interconversion requires an inherently chiral calixarene, whose absolute configuration has been assigned. A crystal is not always readily available for crystal structure determination. CD analysis is limited in inherently chiral calixarene, whose different phenoxyl orientations are confirmatory. The newer DFT calculation seems applicable, but commonly very time-consuming for the too large basis set of inherently chiral calixarenes. The relationship between the absolute configuration of a chiral molecule and its specific optical rotation has long been a very important and well-known research area in stereochemistry. Many empirical and semi-empirical methods and rules, especial those developed by Brewster and Wang [27,28,29,30,31], have been devoted to it. As a part of electron helix theory [29], a variety of approaches have been exhaustively presented by Wang to establish the relationship between absolute configurations of chiral molecules and their helical characters. Their practicability and effectivity are also proven by many simple chiral molecules. However, the helical characters of inherently chiral calixarenes are so complex that their analysis cannot be fully achieved with general approaches in electron helix theory and used to assign their absolute configurations. Although the dilemma exists, we still believe that they are one of the best empirical solutions for the absolute configuration assignment of chiral molecules. Keeping this in mind, we now try to bring forward some approximations and hypothesis in the framework of electron helix theory to achieve the helical electronic energy analysis and absolute configuration assignment of inherently chiral Calix[4]arene.

## 2. Results and Discussion

By keeping the calixarene skeleton integrated, the common dissymmetrical substitutions to create inherent chirality are *O*-alkylation or acylation on the OH group and *meta*- or *para*-substitution on the aromatic ring, although their synthesis can be achieved from either fragment condensation or dissymmetrical derivation of the macrocylic skeleton. Conformational inversion combined with functionalization at different positions can produce a variety of inherently chiral calixarenes [1,2,3,6]. It is known that there are two types of helices: the physical helix originated from asymmetric orbital twisting; and the geometrical helix originated from purely geometrical twisting, in many chiral molecules [29]. Theoretically, these two helices should also exist in inherently chiral Calix[4]arenes.

**Figure 1:**
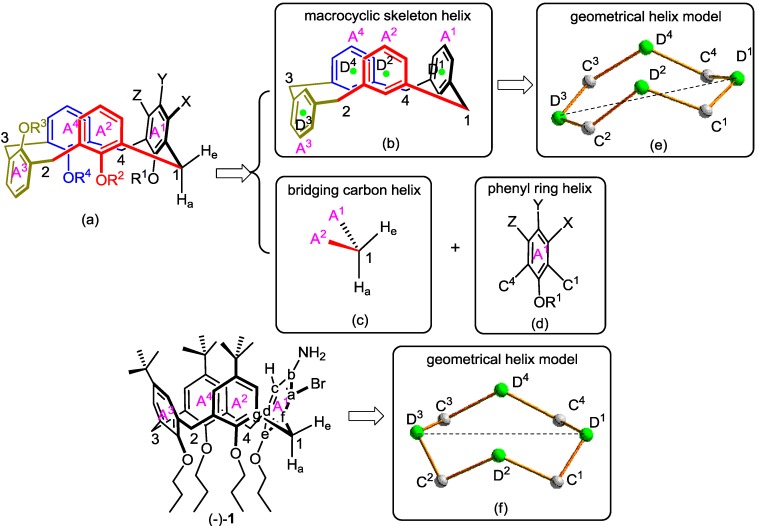
Structural analysis of inherently chiral Calix[4]arene and representative inherently chiral Calix[4]arene (−)-**1**. inherently chiral Calix[4]arene model (**a**); macrocyclic skeleton helix (**b**); bridging carbon helix (**c**); phenyl ring helix (**d**); geometrical helix model of inherently chiral Calix[4]arene model (**e**) and geometrical helix model of (−)-**1** (**f**).

As illustrated in Figure 1, three types of helices, the macrocyclic skeleton helix comprised of four phenyls and four bridging carbons (Figure 1b), the bridging carbon helix originated from asymmetrically substituted bridging carbon *C*^1^ (Figure 1c) and the phenyl ring helix originated from the asymmetrically substituted phenyl ring *A*^1^ (Figure 1d), can be abstracted from the inherently chiral Calix[4]arene model (Figure 1a).

**Table 1:** The dihedral angles and six pairs of exterior angles on phenyl ring *A*^1^ of (−)-**1**.

**Dihedral Angle** **(**°**)**		
*ϕ*_*C*^1^−*C^f^−C^a^−Br*_ = 4.2828	*ϕ_Br−C^a^−C^b^−N_* = 2.8760	*ϕ_N−C^b^−C^c^−N_* = −0.1161
*ϕ*_*N−C^c^−C^d^−C*^4^_ = −6.3989	*ϕ*_*C*^4^−*C^d^−C^e^−O*_ = 6.7100	*ϕ*_*O−C^e^−C^f^−C*^1^_ = −7.0711
**Exterior Angle** **(**°**)**		
*∠BrC^a^C^b^* = 118.450	*∠NC^b^C^a^* = 122.317	*∠HC^c^C^b^* = 119.125
*∠BrC^a^C^f^* = 119.182	*∠NC^b^C^c^* = 119.757	*∠HC^c^C^d^* = 119.096
*∠C*^4^*C^d^C^c^* = 121.734	*∠OC^e^C^d^* = 118.688	*∠C*^1^*C^f^C^e^* = 118.703
*∠C*^4^*C^d^C^e^* = 120.026	*∠OC^e^C^f^* = 118.095	*∠C*^1^*C^f^C^a^* = 124.316

The nature of the sp^2^ hybrid orbital of phenyl carbon can theoretically impel each phenyl and its substituents to be almost located on one plane and their six pairs of exterior angles to be almost 120°, although their substituents can slightly destroy the tendency. For example, in a representative *meta*-substituted inherently chiral Calix[4]arene (−)-**1** [21], the dihedral angles and six pairs of exterior angles on phenyl ring *A*^1^ from its crystal data are shown in Table 1. Since a very large substituent (Br) exists on phenyl ring *A*^1^, these dihedral angles are indeed small and can be omitted, and these exterior angles are all approximately equal to 120°.

In a macrocyclic skeleton helix, four dummy atoms (D^1^, D^2^, D^3^ and D^4^) on four phenyl centers are used to replace four phenyls, respectively. Then, one geometrical helix model (Figure 1e) can be abstracted from the macrocyclic skeleton helix after successively connecting four dummy atoms and four bridging carbons. When the above-stated slight destruction of their substituents is ignored, the geometrical helix model should be equivalent to the macrocyclic skeleton helix. The geometrical helix model can be split into two parts along the dotted line of D^1^D^3^. Since the substitution pattern of all bridging carbons are same, the distances between each dummy atom and bridging carbon should normally be equal. When the relation of the angles exists as *∠D*^1^*C*^1^*D*^2^ = *∠D*^1^*C*^4^*D*^4^, *∠C*^1^*D*^2^*C*^2^ = *∠C*^4^*D*^4^*C*^3^ and *∠D*^2^*C*^2^*D*^3^ = *∠D*^4^*C*^3^*D*^3^, the geometrical helices on the two parts will be canceled. For example, in (−)-**1**, the distances between dummy atoms and bridging carbons and three pairs of angles in the geometrical helix model (Figure 1f) based on its crystal data are shown in Table 2. It is obvious that these distances are indeed approximately equal, and these slight differences between three pairs of angles can indeed be ignored. Therefore, the geometrical helices on its two parts can be canceled, and the whole macrocyclic skeleton helix in (−)-**1** can be omitted.

**Table 2:** The distances between dummy atoms and bridging carbons and three pairs of angles in the geometrical helix model of (−)-**1**.

**Distance** **(**Å**)**					
*d*_*C*^1^*D*^1^_ = 2.9240	*d*_*C*^4^*D*^1^_ = 2.9161		*d*_*C*^1^*D*^2^_ = 2.9212		*d*_*C*^4^*D*^4^_ = 2.9209
*d*_*C*^2^*D*^2^_ = 2.9308	*d*_*C*^3^*D*^4^_ = 2.9385		*d*_*C*^2^*D*^3^_ = 2.9094		*d*_*C*^3^*D*^3^_ = 2.9144
**Angle** **(**°**)**					
*∠D*^1^*C*^1^*D*^2^ = 104.257		*∠C*^1^*D*^2^*C*^2^ = 121.606		*∠D*^2^*C*^2^*D*^3^ = 102.747	
*∠D*^1^*C*^4^*D*^4^ = 108.995		*∠C*^4^*D*^4^*C*^3^ = 121.172		*∠D*^4^*C*^3^*D*^3^ = 102.767	

Four substituents (one equatorial hydrogen (*H_e_*), one axial hydrogen (*H_a_*) and aromatic rings *A*^1^ and *A*^2^) exist on bridging carbon *C*^1^. *H_e_* and *H_a_* differentiate each other due to their outer electronic shielding effect from two adjacent aromatic rings. In all kinds of inherently chiral Calix[4]arenes, the length difference between bond *C*^1^–*H_e_* and *C*^1^–*H_a_*, the angle difference between *∠H_e_C*^1^*A*^1^ and *∠H_a_C*^1^*A*^1^ and the angle difference between *∠H_e_C*^1^*A*^2^ and *∠H_a_C*^1^*A*^2^ are commonly slight and can almost be ignored. For example, in (−)-**1**, the corresponding bond length and angle from its crystal data are as follows: *b*_*C*^1^*H_e_*_ = 0.9904 Å, *b*_*C*^1^*H_a_*_ = 0.9909 Å, *∠H_e_C*^1^*C^f^* = 109.7822°, *∠H_a_C*^1^*C^f^* = 109.8750°, *∠H_e_C*^1^*C^g^* = 109.8749° and *∠H_a_C*^1^*C^g^* = 109.8069°. These slight differences of bond length and angle about *H_e_* and *H_a_* can indeed be ignored. Moreover, until now, the hydrogen polarizability difference originated from different outer electronic environments is still not experimentally or theoretically determined. Here, to simplify the calculation, the above differences of *H_e_* and *H_a_* were tentatively ignored. Then, bridging carbon *C*^1^ (Figure 1a) can be treated as an achiral one. The geometrical helix and physical helix on bridging carbon *C*^1^ can be omitted.

In the phenyl ring helix in Figure 1d, when the above-stated slight destruction of their substituents is ignored, phenyl ring *A*^1^ and its six substituents can be almost treated as coplanar. Then, the geometrical helix on phenyl ring *A*^1^ can be omitted, and only its physical helix needs be considered. Therefore, after the omission of the macrocyclic skeleton helix, bridging carbon helices and geometrical helices on four phenyl rings, only physical helices on four phenyl rings need be considered for the helical character analysis of inherently chiral Calix[4]arenes.

**Figure 2:**
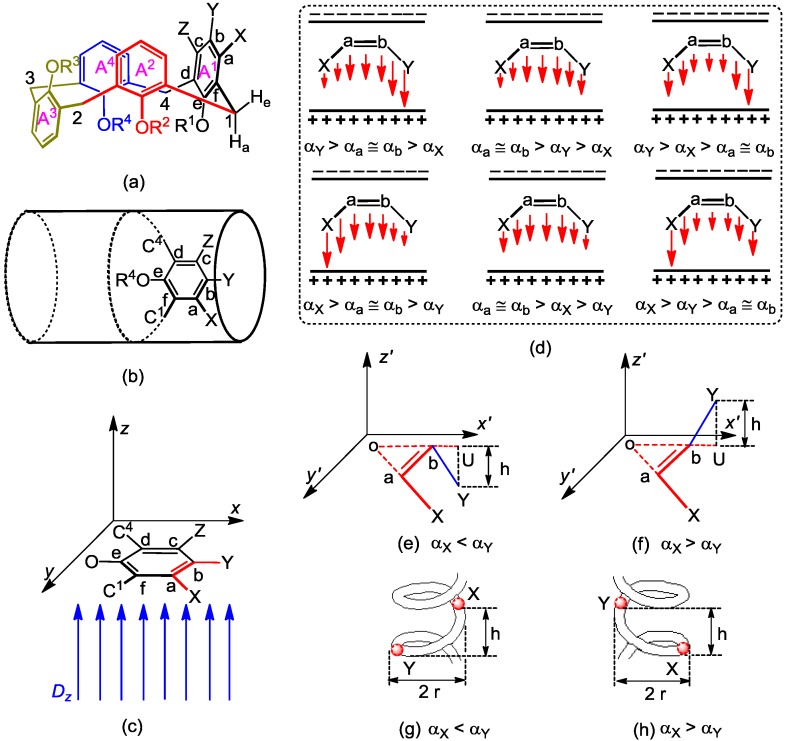
Helical character analysis of microhelix *X–a–b–Y* in inherently chiral Calix[4]arene. inherently chiral Calix[4]arene model (**a**); complex planar chiral model of aromatic ring *A*^1^ (**b**); helical character analysis of aromatic ring *A*^1^ (**c**); different electron movement of bond *Xa*, *ab* and *bY* (**d**); electron movement of bond *bY* relative to *xy* plane when *α_X_* < *α_Y_* (**e**); electron movement of bond *bY* relative to *xy* plane when *α_X_* > *α_Y_* (**f**); right-handed cylindrical microhelix model when *α_X_* < *α_Y_* (**g**) and left-handed cylindrical microhelix model when *α_X_* > *α_Y_* (**h**).

Since all aromatic rings are asymmetric and the architectures on two sides of each aromatic ring plane are different, then inherently chiral Calix[4]arenes can theoretically be regarded as a type of complex planar chiral molecule (Figure 2b). Referring to the helical character analysis for planar chirality [29], the helices of representative aromatic ring *A*^1^ in Figure 2b (the dashed line denotes the remainder of Calix[4]arene) can be resolved into six microhelices, *X–a–b–Y*, *Y–b–c–Z*, *Z–c–d–C*^4^, *C*^4^*–d–e–O*, *O–e–f–C*^1^ and *C*^1^*–f–a–X*, respectively. Here, the microhelical electronic energy of microhelix *X–a–b–Y* was representatively analyzed.

### 2.1. Microhelical Model

The Calix[4]arene cavity is comprised of four aromatic rings and has a high electronic density. Each aromatic ring has a tendency to move away from the cavity to weaken their electrostatic repulsion. As a result, each aromatic ring is essentially equal to being placed in a dissymmetrical electric field. Therefore, the electrons on bonds *Xa*, *ab* and *bY* of microhelix *X–a–b–Y* should move away from their atomic nuclei, and the corresponding induced dipoles would come into being in a dissymmetrical electric field.

Now, a Cartesian coordinate was introduced to analyze the electron movement of bond *bY*, in which the coordinate axis parallel to bond *bY* is set as the *x*-axis, the plane of aromatic ring *A*^1^ is set as the *xy* plane and the coordinate axis perpendicular to the *xy* plane is set as the *z*-axis (Figure 2c). In the orientation parallel to the *x*-axis, since the permanent bonding force between *b* and *Y* is known to be far greater than the normal induced dipole force, the electron movement of bond *bY* parallel to the *x*-axis can be omitted [32]. Moreover, in the orientation parallel to the *y*-axis, since there is two permanent electrostatic repulsions from atom *X* and *Z*, the electron movement of bond *bY* parallel to the *y*-axis can also be omitted. Therefore, only the electron movement of bond *bY* parallel to the *z*-axis needs to be considered. Analogously, only the electron movement of bond *Xa* and *ab* parallel to the *z*-axis need to be considered.

According to the polarizability difference of atom *X*, *a*, *b* and *Y*, the electron movement of bond *Xa*, *ab* and *bY* can be illustrated in Figure 2d, in which electron movement distance is proportional to the length of the arrowed line. If the electron-distorted bonds can be approximately considered as linear and the plane composed of bond *Xa* and *ab* (red bold line) is selected as the *x*'*y*' plane, the electron movement of bond *bY* (blue plain line) relative to the *x*'*y*' plane can be illustrated in Figure 2e,f. When the polarizability sequence is *α_X_* < *α_Y_*, a right-handed cylindrical microhelix model [28,33] can be abstracted in Figure 2g. When the polarizability sequence is *α_X_* > *α_Y_*, a left-handed cylindrical one can be abstracted in Figure 2h.

### 2.2. Microhelical Radius

If the directly connected substituents on all aromatic rings of inherently chiral Calix[4]arene are different, the length of all aromatic bonds and their directly connected bonds should be different. In order to facilitate the calculation of the microhelix radius, one approximation was tentatively made that the length of all aromatic bonds and their directly connected bonds is equal to the average length of all aromatic bonds.

In Figure 2e,f, segment *bU* is the projection of bond *bY* in the *x*'*y*' plane, the point, *o*, is the intersection point of the extension lines of *Xa* and *bU*. Then, based on the approximation, the relative relations of bond length on microhelix *X–a–b–Y* in Figure 2e,f can be drawn as:

*Xa* = *ab* = *bY* = *oa* = *ob* = *B* (1)

here, *B* is a constant, denoting the average length of all aromatic bonds.

The nature of the sp^2^ hybrid orbital of phenyl carbon can theoretically impel each phenyl and its substituents to be almost located on one plane and their six pairs of exterior angles to be almost 120°, which has been proven by a representative *meta*-substituted inherently chiral Calix[4]arene (−)-**1**. Then, the relative relations of angle on microhelix *X–a–b–Y* can be drawn as:

*∠oab = ∠abo = ∠aob = ∠XoU* = 60° (2)

Moreover, in order to facilitate the calculation of the height difference (*h*) between atom *X* and *Y* on the cylindrical microhelix, one hypothesis was artificially brought forward that the electric fields parallel to the *z*-axis (*D_Z_*) acting on all aromatic rings and their directly connected substituents are equal when the conformation of inherently chiral Calix[4]arene becomes stable. Then, the distance (*d*) from the negative charge to the positive charge in the induced dipole moment (*P*) can be calculated as:

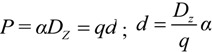
(3)
where *α* is atomic polarizability and *q* is the induced charge. Normally, the bonds between all aromatic rings and their directly connected substituents are the σ bond, in which there are two bonding electrons, one from a substituent and the other from an aromatic carbon. So *q* can be treated as a constant for all microhelices on aromatic rings.

Then, the height difference (*h*) between atom *X* and *Y* on microhelix *X–a–b–Y* can be derived as:

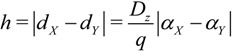
(4)

The length of segment *bU* and *XU* can be derived as:

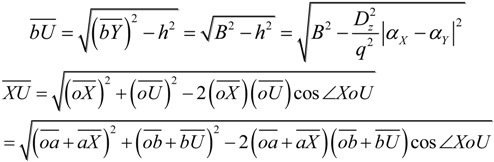
(5)

From Expressions (1), (2) and (5), the length of segment *XU* can be calculated as:

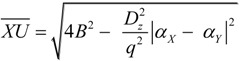
(6)

From sine theorem, the radius (*r*) of microhelix *X–a–b–Y* can be calculated from:

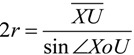
(7)

From Expressions (2) and (6), the microhelical radius can be derived as:

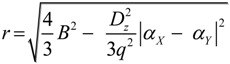
(8)

### 2.3. Microhelical Electronic Energy

The states and eigenvalues of an electron constrained to move on a helix were successfully solved on the basis of the electron-on-a-helix model by Tinoco and Woody [34]. The calculation formula of helical electronic energy (*E*) of a microhelix with an electron of mass *m* constrained on a *k*-turn helix with a radius, *r*, and a pitch, 2π*w*, was deduced as follows:

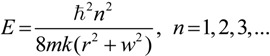
(9)
where *ħ* is the reduced Planck constant and *n* is the quantum number of the transition [31,34]. Apparently, the helical electronic energy (*E*) of microhelix *X–a–b–Y* can theoretically be calculated with the formula.

Since microhelix *X–a–b–Y* is essentially a physical helix and bond *Xa*, *ab* and *bY* are σ bonds, it can be deduced that its pitch should be far less than its radius. Then, for this microhelix, the above calculation formula can be simplified as:

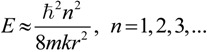
(10)

Then, the reciprocal of electronic energy (*E*) of microhelix *X–a–b–Y* can be deduced from Expression (8) as:

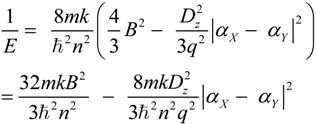
(11)

Since the interior structure and exterior environment of all microhelices on aromatic rings of inherently chiral Calix[4]arene are similar, the variable *k*, *n*, *m* and *q* should be the same to them. Moreover, based on the above approximation and hypothesis, the variables, *B* and *D_Z_* are also the same to them. Then, it can be supposed that:

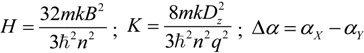
(12)
where *H* and *K* are constant and greater than zero for all microhelices on aromatic rings of inherently chiral Calix[4]arene. Then, Expression (8) can be transformed into:

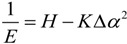
(13)

Essentially, the electronic energy of the electron in a helix is the same whether the helix is right- or left-handed. However, similar to other energy concepts, such as potential energy, the sign of the electronic energy can be artificially stipulated based on a selected reference point. Therefore, in order to distinguish left-handed and right-handed microhelices, we stipulate that Expression (13) is only suitable for a right-handed microhelix and should be changed into Expression (14) when the microhelix is left-handed, as follows:

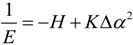
(14)

Actually, the above two expressions can be united into:

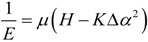
(15)
where *µ* = 1 for the right-handed microhelix and *µ* = −1 for the left-handed microhelix.

By the way, two concepts, “helical character” and “helical electronic energy”, should be tentatively discussed. Wang noted that “in the context of this helix theory, the terms, that is, the helical character and the local energy of electrons of a helix, are equivalent” [30]. From Expression (15) and the illustration in Figure 2, it is obvious that our comprehensive “helical character” comprises helical radius, pitch and orientation, the mass and quantity of electrons constrained on the helix, “helical electronic energy”, exterior electric field acting on the helix, and so on. Therefore, here, the “helical electronic energy” is a connotation of “helical character”, which is slightly different from those proposed by Wang.

There are two rules presented by Wang that “a molecular helix’s helical character (local electronic energy) increases as its length, which usually correlates to its ring size, decreases (Rule I); and, at a fixed helix length, increases as its radius, which correlates to the relevant groups’ polarizability distinctions that result in the bonds’ helical deformations, decreases (Rule II)” [30]. Expression (15) intuitively and clearly shows the impact of helical substituent polarizability distinction on helical electronic energy in inherently chiral Calix[4]arenes, which is actually accordant with Rule II.

Since the above deduction is suitable for all microhelices on aromatic rings of inherently chiral Calix[4]arene, Expression (15) can be universally used to qualitatively calculate their microhelical electronic energy. Therefore, if the sum of the reciprocal of the helical electronic energy of inherently chiral Calix[4]arene is less than zero, it can be assigned as a right-handed helix and dextrorotatory, and *vice versa* [35,36]. Based on Expression (15), we can effectively deduce the signs of the helical electronic energy of inherently chiral Calix[4]arenes and assign their absolute configurations with the limited polarizability data and sequences of atoms directly attached to their microhelices.

**Figure 3:**
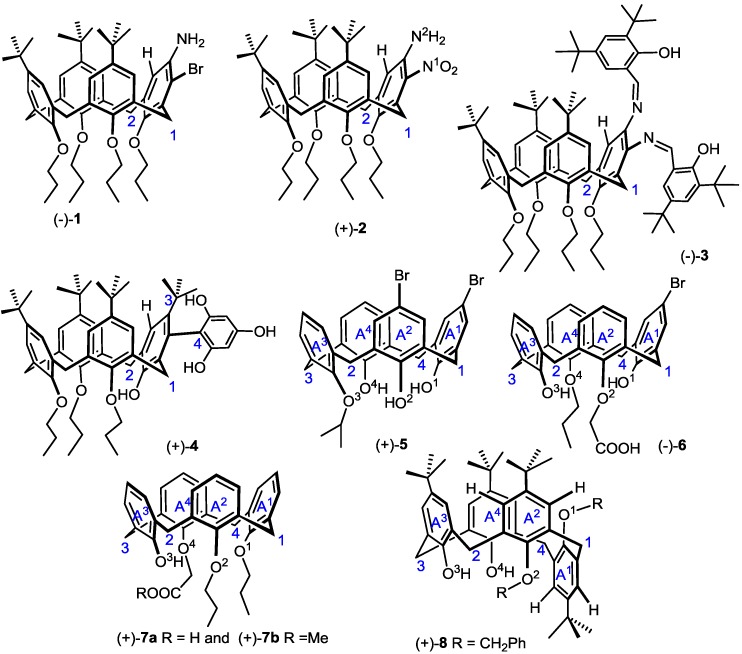
Inherently chiral Calix[4]arenes, whose absolute configuration and optical rotation sign have been ascertained.

Although a variety of inherently chiral Calix[4]arenes were synthesized until now, there are only enumerable entities whose absolute configurations and optical rotation signs have been ascertained. Here, the scientificity of the qualitative calculation of helical electronic energy based on Expression (15) can be verified with inherently chiral Calix[4]arene (−)-**1** [21], (−)-**2** [21], (−)-**3** [22], (+)-**4** [26], (+)-**5** [23], (−)-**6** [12], (+)-**7a** and (+)-**7b** [24] and (+)-**8** [25] (Figure 3). Prior to the qualitative calculation of their helical electronic energy, the polarizability of their bridging carbons should be differentiated. Each bridging carbon is connected with one equatorial hydrogen (*H_e_*), one axial hydrogen (*H_a_*) and two different aromatic rings. The difference of *H_e_* and *H_a_* can be also ignored as stated above. Then, the bridging carbon polarizability should be decided by the electrification of the two remaining aromatic rings.

In (−)-**1**, its atom polarizability sequence on the *meta*-substituted aromatic ring is *α_Br_* > *α*_*C*^1^_ ≈ *α*_*C*^2^_ > *α_N in NH_2__* > *α_H_* and (*α_Br_* (3.013) + *α_H_* (0.387)) > (*α*_*C*^1^_ (1.061) + α_N in NH_2__ (0.964)) [37]. Then, without consideration of microhelices canceled by each other, the reciprocal of its helical electronic energy can be deduced from microhelices *C^1^–Br*, *Br–N*, *N–H* and *C^2^–H* (here, the microhelix is simply illustrated with one dashed line and two atomic labels on its two ends) as:

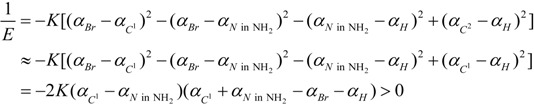
(16)

Therefore, it should be a left-handed helix and levorotatory.

In (+)-**2**, its atom polarizability sequence on the *meta*-substituted aromatic ring is *α*_*N* in NO_2__ > *α*_*C*^1^_ ≈ *α*_*C*^2^_ > *α*_*N* in NH_2__ > *α_H_* and (*α*_*N* in NO_2__ (1.090) + *α_H_* (0.387)) < (*α*_*C*^1^_ (1.061) + *α*_*N* in NH_2__ (0.964)) [37]. Then, without consideration of microhelices canceled by each other, the reciprocal of its helical electronic energy can be deduced from microhelices *C^1^–N^1^*, *N^1^–N^2^*, *N^2^–H* and *C^2^–H* as:

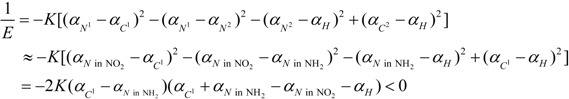
(17)

Therefore, it should be a right-handed helix and dextrorotatory.

In (−)-**3**, its atom polarizability sequence on the *meta*-substituted aromatic ring is *α_N in N=C_* > *α*_*C*^1^_ ≈ *α*_*C*^2^_ > *α_H_* [37]. Then, without consideration of microhelices canceled by each other, the reciprocal of its helical electronic energy can be deduced from microhelices *C^1^–N*, *N–H* and *C^2^–H* as:

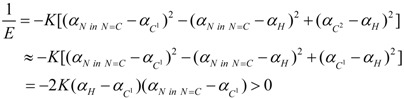
(18)

Therefore, it should be a left-handed helix and levorotatory.

In (+)-**4**, its atom polarizability sequence on the *meta*-substituted aromatic ring is *α*_*C* in Ar_ > *α*_*C*^1^_ ≈ *α*_*C*^2^_ ≥ *α*_*C* in CM_e_3___ > *α_H_* (here, Ar denotes 2,4,6-trihydroxyphenyl) and (*α*_*C* in Ar_ (1.352) + *α_H_* (0.387)) > (*α*_*C*^1^_ (1.061) + *α*_*C *in CM_e_3___ (1.061)) [37]. Then, without consideration of microhelices canceled by each other, the reciprocal of its helical electronic energy can be deduced from microhelices *C^1^–C^4^*, *C^4^–C^3^*, *C^3^–H* and *C^2^–H* as:


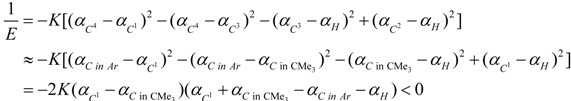
(19)

Therefore, it should be a right-handed helix and dextrorotatory.

In (+)-**5**, since the electron-withdrawing capability is *Br* > *H* and *OCH*(*CH*_3_)_2_ > *OH*, the phenyl polarizability should be *α*_*A*^4^_ > *α*_*A*^3^_ >> *α*_*A*^2^_ ≈ *α*_*A*^1^_ and the bridging carbon polarizability should be *α*_*C*^3^_ > *α*_*C*^2^_ ≈ *α*_*C*^4^_ >> *α*_*C*^1^_ (because the polarizability is electron-rich groups > electron-poor analogues) [29]. Moreover, since the bond polarizability is *O* − *H* ˃ *O* − *C* [37], the oxygen polarizability should be *α*_*O*^1^_ ≈ *α*_*O*^2^_ ≈ *α*_*O*^4^_ > *α*_*O*^3^_. Then, besides those symmetrical microhelices, microhelices *C^2^–O^2^* and *C^4^–O^1^* and microhelices *C^1^–O^2^* and *C^1^–O^1^* can also be canceled by each other. Without consideration of microhelices canceled by each other, the reciprocal of its helical electronic energy can be deduced from microhelices *C^3^–O^3^*, *C^2^–O^3^*, *C^3^–O^4^* and *C^4^–O^4^* as:

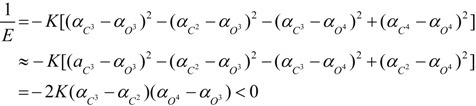
(20)

Therefore, it should be a right-handed helix and dextrorotatory.

In (−)-**6**, since the electron-withdrawing capability is *Br* >> *H* and *O*^4^ *Pr* ≈ *O*^2^*CH*_2_*COOH* > *O*^3^*H* ≈ *O*^1^*H*, the phenyl polarizability should be *α*_*A*^3^_ > *α*_*A*^4^_ ≈ *α*_*A*^2^_ >> *α*_*A*^1^_ and the bridging carbon polarizability should be *α*_*C*^3^_ ≈ *α*_*C*^2^_ >> *α*_*C*^4^_ ≈ *α*_*C*^1^_. Moreover, since the polarizability sequences are *O* − *H* ˃ *O* − *C* and *α*_carbonyl carbon_ > *α*_alkyl carbon_ [37], the oxygen polarizability is *α*_*O*^3^_ ≈ *α*_*O*^1^_ > *α*_*O*^2^_ > *α*_*O*^4^_. Then, besides those symmetrical microhelices, microhelices *C^1^–O^1^* and *C^4^–O^1^* and microhelices *C^2^–O^3^* and *C^3^–O^3^* can also be canceled by each other. Without consideration of microhelices canceled by each other, the reciprocal of its helical electronic energy can be deduced from microhelices *C^2^–O^2^*, *C^1^–O^2^*, *C^4^–O^4^* and *C^3^–O^4^* as:

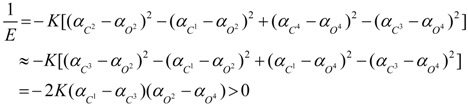
(21)

Therefore, it should be a left-handed helix and levorotatory.

In (+)-**7a** and (+)-**7b**, since the electron-withdrawing capability is *O*^4^*CH*_2_*COOH* ≥ *O*^2^*Pr* ≈ *O*^1^*Pr* > *O*^3^*H* (for (+)-**7a**) and *O*^4^*CH*_2_*COOH* ≥ *O*^2^*Pr* ≈ *O*^1^*Pr* > *O*^3^*H* (for (+)-**7b**), their phenyl polarizability should be *α*_*A*^3^_ > *α*_*A*^2^_ ≈ *α*_*A*^1^_ ≥ *α*_*A*^4^_ and the bridging carbon polarizability should be *α*_*C*^3^_ ≥ *α*_*C*^2^_ > *α*_*C*^1^_ ≥ *α*_*C*^4^_. Moreover, since the polarizability sequences are *O* − *H* ˃ *O* − *C* and *α*_carbonyl carbon_ > *α*_alkyl carbon_ [37], the oxygen polarizability is *α*_*O*^3^_ > *α*_*O*^4^_ > *α*_*O*^2^_ ≈ *α*_*O*^1^_. Then, besides those symmetrical microhelices,microhelices *C^1^–O^2^* and *C^1^–O^1^* can also be canceled by each other. Without consideration of microhelices canceled by each other, the reciprocal of their helical electronic energy can all be deduced from microhelices *C^2^–O^3^*, *C^2^–O^2^*, *C^3^–O^4^*, *C^3^–O^3^*, *C^4^–O^1^* and *C^4^–O^4^* as:

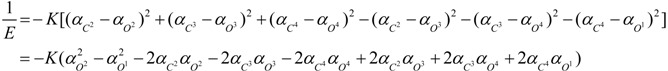
(22)

Since *α*_*C*^3^_ ≥ *α*_*C*^2^_ and *α*_*O*^2^_ ≈ *α*_*O*^1^_, then:

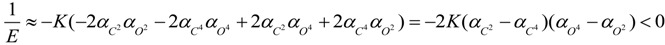
(23)

Therefore, they should be right-handed helices and dextrorotatory.

In (+)-**8**, since the electron-withdrawing capability is *O*^1^*CH*_2_*Ph* ≈ *O*^2^*CH*_2_*Ph* > *O*^3^*H* ≈ *O*^4^*H* [29], the phenyl polarizability should be *α*_*A*^3^_ ≈ *α*_*A*^4^_ > *α*_*A*^1^_ ≈ *α*_*A*^2^_ and the bridging carbon polarizability should be *α*_*C*^3^_ > *α*_*C*^2^_ ≈ *α*_*C*^4^_ > *α*_*C*^1^_. Moreover, since the group polarizabilities are *O* − *H* ˃ *O* − *C*, the oxygen polarizability should be *α*_*O*^3^_ ≈ *α*_*O*^4^_ > *α*_*O*^1^_ ≈ *α*_*O*^2^_. Then, besides those symmetrical microhelices, microhelices *C^3^–O^3^* and *C^3^–O^4^*, microhelices *C^2^–O^3^* and *C^4^–O^4^* and microhelices *C^2^–H* (on aromatic ring *A*^3^) and *C^4^–H* (on aromatic ring *A*^4^) can also be canceled by each other. Without consideration of microhelices canceled by each other, the reciprocal of its helical electronic energy can be deduced from microhelices *C^2^–H*, *C^2^–O^2^*, *C^1^–O^2^* and *C^1^–H* on aromatic ring *A*^2^ and *C^1^–H*, *C^1^–O^1^*, *C^4^–O^1^* and *C^4^–H* on aromatic ring *A*^1^ as:

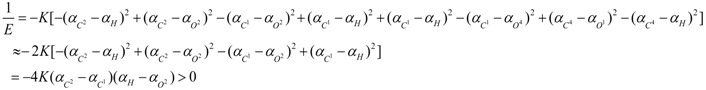
(24)

Therefore, it should be a left-handed helix and levorotatory.

It should be mentioned that geometrical helices, resulting from an intermolecular or intramolecular non-bonded interaction in a high concentration and polar solvents, are not taken into consideration in the above analysis and calculation from Expression (15). Therefore, the calculated results only can be compared with those measured in low concentration and non-polar solvents. It is very surprising and satisfying that all of optical rotations from the above analysis are consistent with the actual facts, except (+)-**8**. Therefore, from the above case analysis, the qualitative analysis of the helical electronic energy of inherently chiral Calix[4]arenes with Expression (15) is almost entirely proven as scientific and can be used to effectively assign their absolute configurations.

Due to structural similarity, Expression (15) can be popularized to assign the absolute configurations of other inherently chiral calix[*n*]arenes (*n* = 5, 6, 8) and other inherently chiral concave molecules. However, it must be admitted that this expression is only a qualitative analysis tool for the helical electronic energy of inherently chiral Calix[4]arenes. The above approximation and hypothesis in its deduction need to be further verified, and the variables, *m*, *q* and *D*_Z_, need to be quantified with theoretic deduction and experimental data. Moreover, the exception from (+)-**8** may be satisfactorily interpreted in an upcoming quantificational expression. Therefore, the relevant verification and variable quantification will be explored in our subsequent works.

## 3. Conclusions

In summary, inherently chiral Calix[4]arenes can be theoretically regarded as a type of complex planar chiral molecule when bridging carbons are treated as achiral and each phenyl ring and its six substituents are treated as coplanar. Based on one approximation and one hypothesis, we derive Expression (15) to qualitatively analyze microhelical electronic energy. Its scientificity and effectivity in absolute configuration assignments of inherently chiral Calix[4]arenes were almost entirely confirmed with all of the entities, whose absolute configurations and optical rotation signs have been ascertained.
